# Interrelationship Between Physical Exercise and Apical Periodontitis in Trained Rats

**DOI:** 10.1155/ijod/7317709

**Published:** 2026-01-02

**Authors:** Hernán Coaguila-Llerena, Elda Olivia Nobre de Souza, Ana Beatriz Bardasi Solcia, Gilmara Gomes de Assis, Sandra Lia do Amaral, Paulo Sérgio Cerri, Gisele Faria

**Affiliations:** ^1^ School of Dentistry, Department of Restorative Dentistry, São Paulo State University (UNESP), Araraquara, São Paulo, Brazil, unesp.br; ^2^ School of Stomatology, Department of Endodontics, Cayetano Heredia Peruvian University (UPCH), Lima, Peru, cayetano.edu.pe; ^3^ School of Sciences, Department of Physical Education, São Paulo State University (UNESP), Bauru, São Paulo, Brazil, unesp.br; ^4^ School of Dentistry, Department of Morphology, Genetics, Orthodontics and Paediatric Dentistry, Laboratory of Histology and Embryology, São Paulo State University (UNESP), Araraquara, São Paulo, Brazil, unesp.br

## Abstract

**Objective:**

To assess the influence of physical exercise on apical periodontitis (AP) development, and the effect of AP on physical exercise capacity in exercise‐trained rats.

**Materials and Methods:**

After animal Ethics Committee approval, 40 male Holtzman rats (9 weeks old) were distributed into the following four groups (*n* = 10/group): physical exercise without AP induction (Ex); physical exercise with AP induction (Ex‐AP); sedentary without AP induction (S); sedentary with AP induction (S‐AP). In trained groups, animals performed moderate‐intensity aerobic training (treadmill) for 11 weeks. The maximal capacity test (*T*_max_) was performed at baseline (0 weeks), 8 and 11 weeks to analyse physical capacity and increase training velocity to maintain training intensity. In the AP‐induced groups, coronal access of the left maxillary and mandibular first and second molars was performed after 8 weeks of training, and the pulp chamber was kept open for 21 days. After euthanasia, the hemi‐mandible was submitted to histopathological, radiographical and micro‐computed tomography (micro‐CT) analyses to evaluate the inflammation, the area and the volume of periapical bone resorption, respectively.

**Results:**

The *T*_max_ results of trained groups were higher than those of sedentary groups (*p* < 0.05). There was no difference in *T*_max_ results between trained groups (Ex and Ex‐AP; *p* > 0.05), or between sedentary groups (S and S‐AP) (*p* > 0.05). Regarding volume and area of apical bone resorption, there was no difference between S‐AP and Ex‐AP (*p* > 0.05). Histopathological qualitative analysis also showed no differences between S‐AP and Ex‐AP.

**Conclusion:**

Physical exercise did not influence AP development, nor did AP affect physical exercise capacity in trained rats.

## 1. Introduction

Apical periodontitis (AP) is a disease with a high worldwide prevalence [1]. AP is a localised inflammatory response resulting from the progression of microorganisms or their products in the root canal system, wherein an immuno‐inflammatory response mediates the destruction of the periodontal ligament, alveolar bone and dental tissues (cementum and dentine) [2–4]. The inflammatory response involves the activation of endothelial cells, neutrophils, macrophages, lymphocytes, osteoclasts, dendritic cells and fibroblasts [5, 6]. This activation leads to the production of several pro‐inflammatory cytokines, including interleukin 1β (IL‐1β), IL‐6 and tumour necrosis factor alpha (TNF‐α), as well as upregulation of receptor activator of NF‐κB ligand (RANKL) and matrix metalloproteinases (MMPs), which together promote osteoclastogenesis and bone resorption [2, 7]. Thus, the development and evolution of AP are affected by the host’s immuno‐inflammatory response [8].

Certain systemic conditions may have implications for the development and progression of AP. A study revealed that rats with liver fibrosis had AP exacerbation, high levels of IL‐1β, IL‐6 and TNF‐α, and increased inflammatory infiltrate and periapical bone resorption [9]. Diabetes mellitus has also been found to accelerate AP development and progression [10]. On the other hand, AP can influence systemic conditions, given that it increases the number of neutrophils, and lymphocyte and blood glucose concentrations in rats with diabetes mellitus [10]. Furthermore, AP treatment can improve the systemic oxidative condition induced by diabetes [11]. For this reason, the link between endodontics and systemic health cannot be ignored by professionals in their clinical practice [12]. Although there is evidence associating AP with different systemic health‐related parameters, very little is known about the impact of AP on an individual’s physical exercise performance. Indeed, only one ’preliminary’ study [13] in the literature showed that higher AP levels in patients with periodontal disease were associated independently with poor physical fitness [13]

On the other hand, any physical activity, for example, physical exercise, is associated with improved health and quality of life [14]. Although physical exercise is generally linked to a positive impact on weight loss [15], its overall benefits apply to all body systems, including musculoskeletal, respiratory, hormonal and immune [16].

The literature has shown that lower concentrations of inflammatory markers are observed in individuals who perform physical exercise [17]. Specifically, an acute increase in IL‐6 and IL‐10 levels induced by physical exercise has direct anti‐inflammatory effects by inhibiting TNF‐α, and by stimulating IL‐1ra, thereby limiting IL‐1β signalling [18, 19]. Indirect long‐term anti‐inflammatory effects mediated by limiting abdominal adiposity have been found to improve body composition, which affects chronic systemic inflammation [18]. Studies in dentistry have shown that regular physical activity has beneficial effects on reducing the prevalence of periodontal disease and disease‐related inflammatory cytokines, and in promoting periodontal health [20–22]. This effect can be attributed to the production and modulation of inflammatory mediators promoted by physical exercise [20, 22].

Considering the pathogenesis of AP, physical exercise could attenuate tissue destruction in an AP‐inflamed environment, given that it is a modulating agent of the host’s inflammatory response. A recent study revealed that rats taking on physical exercise at the same time as AP induction developed a smaller periapical lesion (AP) than those remaining sedentary [23]. However, there is no evidence that physical exercise affects AP development in individuals with a history of physical exercise, that is, under training. Additionally, to the best of our knowledge, there is no evidence that AP influences physical exercise capacity. Thus, this study aimed to assess the effect of physical exercise on AP development, and the impact of AP on physical exercise capacity in trained rats. The null hypothesis is that exercise does not affect AP development, and vice versa.

## 2. Materials and Methods

### 2.1. Animals

The experimental procedures were approved by the Animal Ethics Committee of the school of dentistry (Approval No. 20/2022). The study is reported following ARRIVE guidelines [24]. The study used 40 male Holtzman rats (*Rattus norvegicus albinus*), 9 weeks old, weighing an average 190–200 g, obtained from the central vivarium of the university. Animal care was performed by four trained operators (Hernán Coaguila‐Llerena, Elda Olivia Nobre de Souza, Ana Beatriz Bardasi Solcia and Gilmara Gomes de Assis), which were monitored by a veterinary technician, and supervised by a certified veterinarian. The animals were kept in polypropylene cages with perforated stainless‐steel lids (three animals per cage), lined with wood shavings, which was replaced every 3 days. Temperature (22 ± 2°C) and relative humidity (55% ± 10%) were constant, on a 12:12 h light–dark cycle, with a standard laboratory diet and water ad libitum.

### 2.2. Sample Size Calculation

The sample size was estimated using 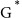
Power (v3.1.9.7), considering a one‐way ANOVA with four independent groups, *α* = 0.05 significance level, and 80% statistical power (1 – *β* = 0.80). A moderate‐to‐large effect size (*f* = 0.6) [25] was adopted, based on preliminary data from our previous pilots, and on sample sizes used in similar published studies [23, 26]. The calculation showed that nine animals were needed per group. However, considering that losses could occur during the anaesthesia procedure (animal death) or surgery (tooth fracture or perforation), 10 animals per group were used for 12 weeks (1 week for adaptation and 11 experimental weeks). To set experimental groups, animals were randomly allocated.

### 2.3. Implementation of Physical Exercise

**Figure 1:**
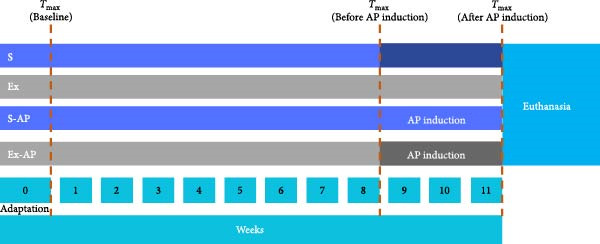
Illustration of the experimental design for 11 weeks of physical exercise without AP induction (Ex); physical exercise with AP induction (Ex‐AP); sedentary without AP induction (S); sedentary with AP induction (S‐AP). Maximal capacity test (*T*_max_).

As seen in Figure 1, the animals were subjected to an adaptation period of 5 min per day, for 7 days, on a specific treadmill for rodents, at a speed of 3–6 m/min. Then, the animals were subjected to a maximal capacity test (*T*_max_) following a previous protocol [27], which consisted of running on a treadmill, 0% grade, with 5 m/min increments every 3 min, until the animal stopped running spontaneously. The *T*_max_ was used to prescribe the moderate‐intensity aerobic training protocol, as previously described [27–29]. After the first *T*_max_, animals that had been previously coded were randomly assigned to the experimental groups by drawing their identification numbers from an opaque envelope. The four groups (*n* = 10/group) were: physical exercise without AP induction (Ex); physical exercise with AP induction (Ex‐AP); sedentary without AP induction (S); sedentary with AP induction (S‐AP). The physical exercise protocol was carried out in two consecutive experimental stages: (1) initially, 8 weeks of training (or remaining sedentary, in the case of untrained groups); (2) and then, 3 weeks (21 days) of AP induction by keeping the pulp chamber open to the oral environment. The animals kept training (or remained sedentary, in the case of untrained groups) for a total of 11 experimental weeks. All the groups were subjected to *T*_max_ at time point 0 (experiment baseline), at 8 weeks (before AP induction) and at 11 weeks (after AP induction) to analyse physical capacity and increase training velocity to maintain training intensity. An additional 4‐week *T*_max_ was performed to adjust training velocity to maintain training intensity. In the Ex groups, the animals underwent moderate‐intensity aerobic training using treadmill running for the rats (0% grade), with an intensity of 60% of the *T*_max_, 1 h per day, 5 days/week [28], for 11 weeks. The running time taken by the animals at each *T*_max_ was recorded in seconds. In the groups with AP induction, training began 2 days after performing coronal access, because the animals were subjected to general anaesthesia for this procedure. The animals were weighed weekly throughout the experiment to monitor any possible alterations, and the data are presented for the ’baseline’, ’before’ and ’after’ AP induction time points.

### 2.4. Induction of AP

AP induction was performed in the 8^th^ week of the experiment (Figure 1), according to a previous protocol [30]. The rats were anaesthetized intraperitoneally by intramuscular injection of 10% ketamine hydrochloride (80 mg/kg) and 2% xylazine (7 mg/kg), placed in a supine position, and positioned on a jaw retraction device. All efforts were made to minimise suffering. Coronal access of the left first and second molars was made with a # ¼ stainless‐steel spherical drill (KaVo, Brea, CA, USA), mounted on an electric motor (Endo ProTorque, Driller, Carapicuíba, SP, Brazil). Postoperative animal care after coronal access included: injection with a single dose of sodium dipyrone, 150 mg/kg (Febrax, Lema Injex Biologic, Vespasiano, MG, Brazil) to control pain and discomfort, and monitored rest with soft food for 2 days. Subsequently, the animals continued aerobic physical training, as described above. The pulp chamber was kept open to the oral environment for 21 days to induce AP formation. Afterward, the animals were euthanized by anaesthetic overdose of ketamine hydrochloride and xylazine, the left hemi‐mandibles were collected, fixed in 4% buffered formaldehyde (prepared from paraformaldehyde) for 72 h and submitted to radiographical, microcomputed tomographical and histopathological analyses to evaluate the inflammation, the area and the volume of periapical bone resorption, respectively. The radiographical analysis was complemented with microcomputed tomographical analysis to assess possible differences between AP area and volume.

### 2.5. Radiographical Analysis

The left hemi‐mandibles were radiographed in a standard position (perpendicular angle between buccal molar surface and X‐ray beam, at 10 cm focal distance) using an X‐ray device (Spectro 70X unit, Dabi Atlante, Ribeirao Preto, SP, Brazil) operated at 90 kV and 0.20 s exposure time, and a digital radiography system (Kodak RVG 6100, Kodak Dental Systems, NY, USA). The area (mm^2^) of the apical periodontal space (groups without AP induction) and that of the apical bone resorption (groups with AP induction) were measured concerning the distal root of the first and second mandibular molars, by using ImageJ software (National Institutes of Health). The measurements were performed by a blinded trained examiner.

### 2.6. Micro‐Computed Tomography (Micro‐CT) Analysis

The hemi‐mandibles were scanned using a micro‐CT device (Phoenix v|tome|x s240, General Electric, Boston, MA, USA) with 1000 projections, skipping 1, averaging 3, exposure time 200 ms, voltage 70 kV, current 200 μA, magnification 11.1–13.3x, voxel size 15.0–18.0 μm and a 0.1 mm copper filter. Reconstructions were performed with Datos|x 2 software (General Electric, Boston, MA, USA). DataViewer software (Skyscan, Kontich, Belgium) aligned the images in the coronal, axial, and sagittal planes [31]. Afterward, the sagittal plane images were transferred to CTAn v.1.15.4.0 software (Skyscan) to measure the periodontal apical space volume (groups without AP induction) and the apical bone resorption (groups with AP induction) of the distal roots, in mm^3^. The measurements were performed by a blinded trained examiner.

### 2.7. Inflammation Analysis

After microtomographic scanning, the hemi‐mandibles were decalcified in an EDTA‐based solution, subjected to the routine histological technique, and embedded in paraffin. The specimens were sectioned) longitudinally in the buccolingual direction. Serial sections of 5 µm thickness were obtained. The histopathological analysis was conducted by staining the sections with haematoxylin‐eosin (H&E) and evaluating them under a conventional light microscope (Olympus BX51, Tokyo, Japan). A qualitative analysis of the periapical region (including root apex, dental pulp, cementum, periodontal ligament and adjacent alveolar bone) was conducted on the distal roots. The parameters for qualitative analysis were: presence of inflammatory infiltrate (absent and present), condition of the dental pulp (healthy and necrotic), condition of the cementum (normal and resorbed) and the width of the periodontal ligament space (normal/narrow, widened but without detectable bone resorption, detectable apical bone resorption and severe apical bone resorption).

### 2.8. Statistical Analysis

The data were processed using GraphPad Prism 9 Software (GraphPad Software, San Diego, CA, USA) at a 5% significance level. The apical bone resorption data (periapical lesion) had normal distribution, as confirmed by the Shapiro–Wilk test; however, the standard deviations were significantly different (Bartlett’s test). Therefore, the non‐parametric Kruskal–Wallis test and Dunn’s post hoc test were applied.

## 3. Results

There was no loss of any animal during the experiment.

### 3.1. Weight and Physical Exercise Capacity

There was no weight loss in any of the animals of any group during the entire experimental period. At baseline and 8 weeks (before AP induction), no significant differences were observed among the groups (*p* > 0.05). 3 weeks after AP induction (after 11 weeks), the weight of the trained groups (Ex and Ex‐AP) was higher than that of the sedentary groups (S and S‐AP; *p* < 0.05) (Figure 2).

Figure 2Weight change during the experimental period expressed in g (a). Treadmill running time during maximal capacity tests (*T*_max_) (b). ^#^Significant difference between trained groups (Ex and Ex‐AP) and sedentary groups (S and S‐AP).  ^∗^Significant difference versus baseline (*p* < 0.05).

**Figure:**
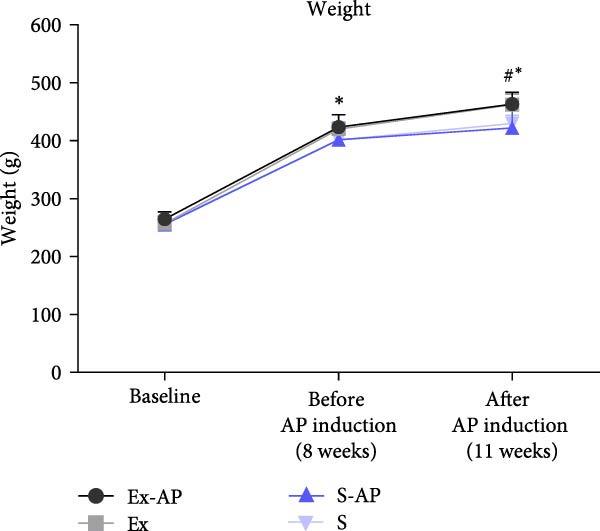
(a)

**Figure:**
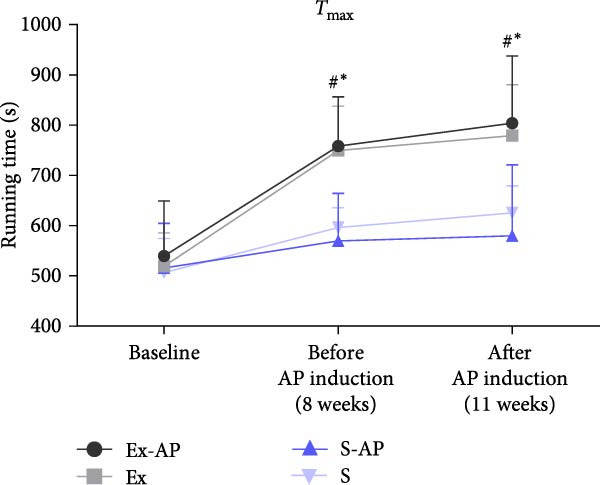
(b)

Before beginning the exercise protocol, there were no differences in the *T*_max_ results, among any of the groups (*p* > 0.05). The training protocol improved the *T*_max_ score. At 8 weeks (before AP induction), the *T*_max_ score for the trained groups was higher than that of the sedentary groups (*p* < 0.05). This difference was maintained at 11 weeks (3 weeks after AP induction; *p* > 0.05). The application of AP did not influence the animals’ *T*_max_ score, given that there was no difference in the *T*_max_ result between the trained and sedentary groups (*p* > 0.05; Figure 2) at the end of the protocol.

### 3.2. Radiographical and Micro‐CT Analyses

Physical exercise did not influence AP development, given that there was no significant difference regarding the area or volume of apical bone resorption between the S‐AP and Ex‐AP groups, in either the first or the second molars (*p* > 0.05; Figure 3).

Figure 3Quantitative comparative analysis of bone resorption area (a) and volume (b) in left mandibular first and second molars. Different lowercase letters in columns, corresponding to each tooth, indicate significant differences (*p* < 0.05). Representative illustrations of radiographical (c) and micro‐CT (d) analyses.

**Figure:**
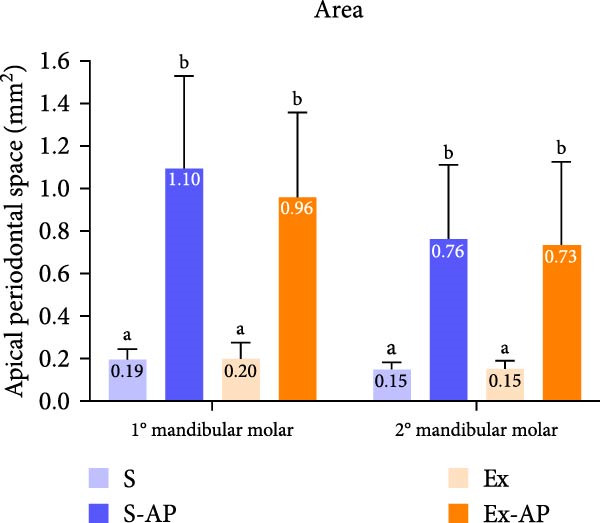
(a)

**Figure:**
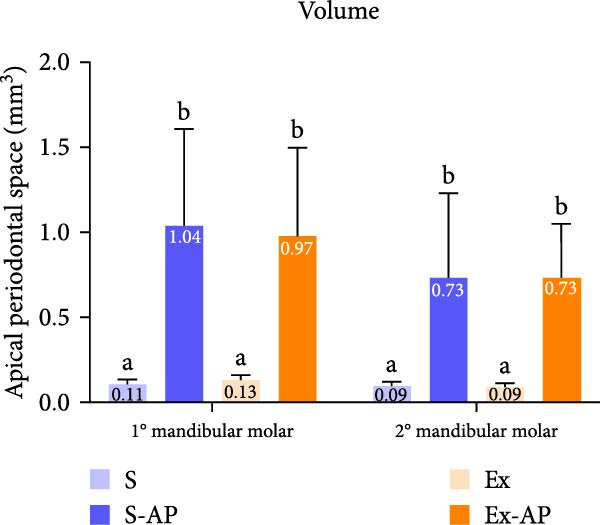
(b)

**Figure:**
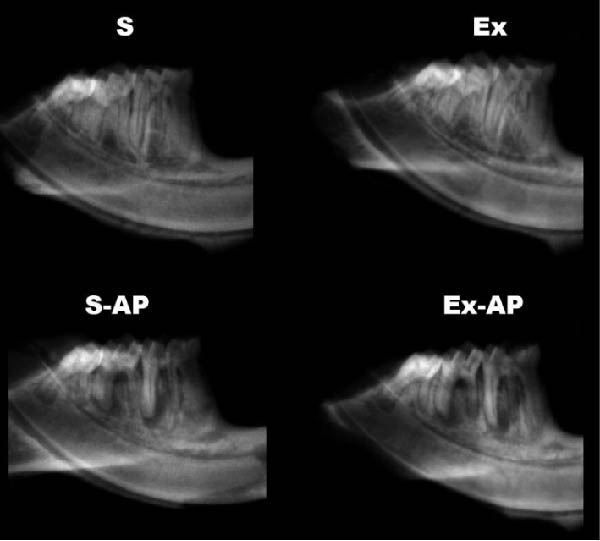
(c)

**Figure:**
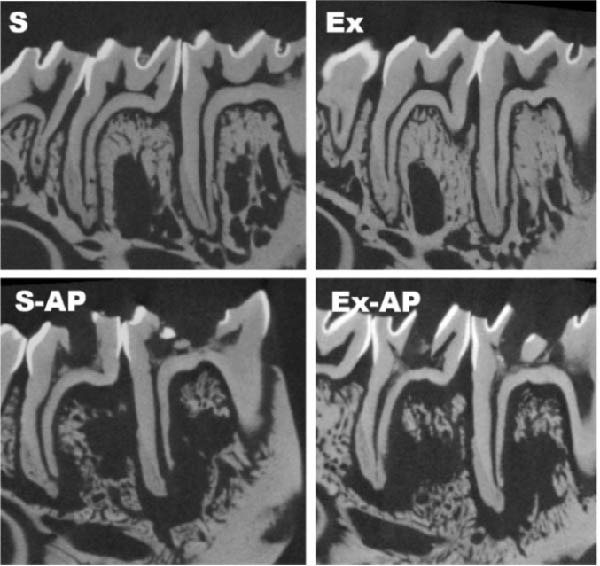
(d)

### 3.3. Histopathological Analysis

Histopathological analysis of the first and second molars showed that, in the groups with AP induction, whether sedentary or trained, there was no pulp area (healthy dental pulp), the periodontal ligament was unstructured, and there was bone and cementum resorption, as well as inflammatory infiltrate, composed mainly of polymorphonuclear neutrophils near the apex and mononuclear cells in the rest of the AP regions. The groups without AP induction showed no alterations in the pulp, apex or surrounding tissues (i.e., the pulp was normal with an odontoblastic and predentine layer, no alterations in the cementum or periodontal ligament and alveolar bone integrity; Figure 4).

Figure 4Light micrographs of portions of mandible showing distal root (R) surrounded by periodontal tissues. (a) (S) and (c) (Ex): root canals are filled by pulp tissue (DP) which is continuity with healthy periodontal ligament (PL). Alveolar bone process (AB) surrounds the narrow periodontal space. (b) (S‐AP) and (d) (Ex‐AP): necrotic pulp remains (NP) fill partially the root canal. Dense infiltrated inflammatory (IC) is observed in close juxtaposition to the apical foramen. The periodontal space (PS) is enlarged and surrounded by small and thin bone trabeculae (AB). H&E. Scale bars: 370 µm.

**Figure:**
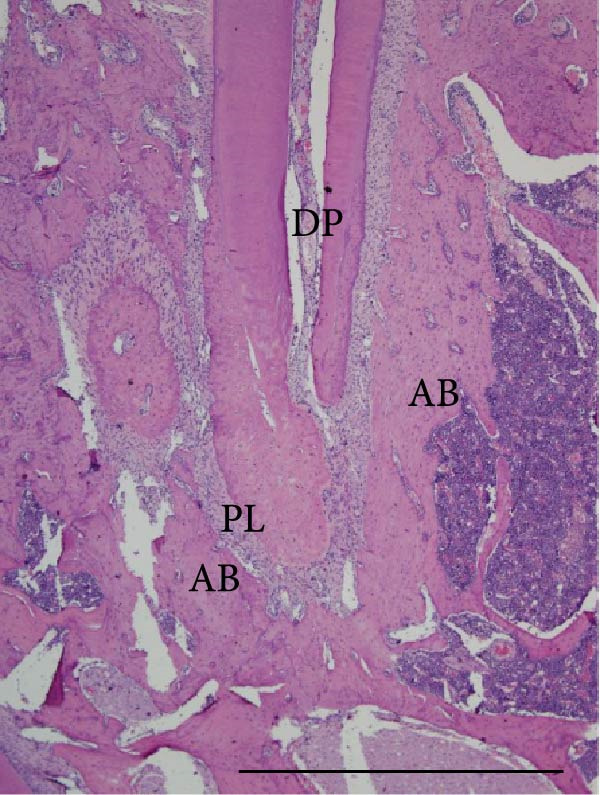
(a)

**Figure:**
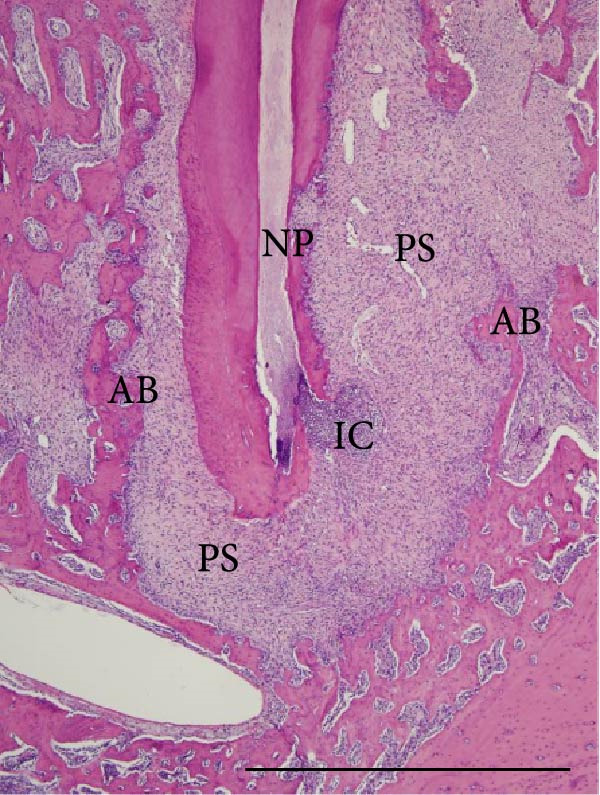
(b)

**Figure:**
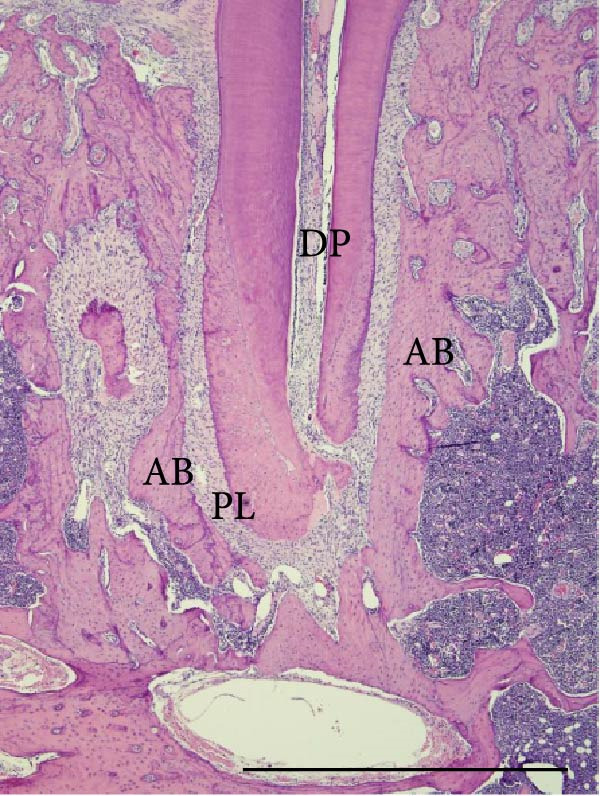
(c)

**Figure:**
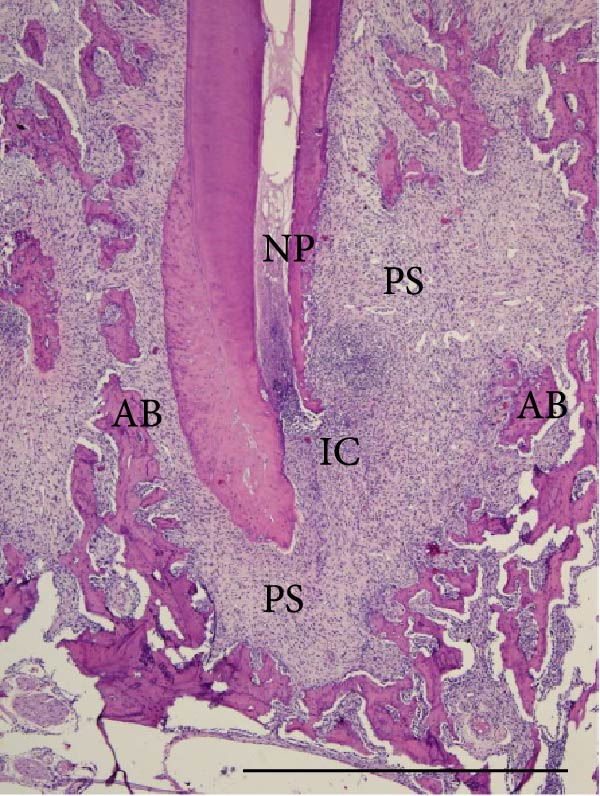
(d)

## 4. Discussion

This study assessed the influence of physical exercise on AP development and, conversely, the impact of AP on exercise performance in trained rats. The null hypothesis was accepted because no differences were observed between the trained groups (Ex and Ex‐AP) and the sedentary groups (S and S‐AP), that is, AP did not affect physical exercise capacity, or vice versa.

AP induction was performed in a rat model widely used in endodontic and physical exercise research [23, 28–30]. The advantage of this model is that standardised conditions can be established, such as the same lineage, sex, weight, food, stress and accommodation, that is, an option that would be impractical in a human model [32]. In the present study, a 21‐day AP development period was chosen because this duration represents the end of the AP development active stage [33]. There are two stages in the formation of experimentally induced AP: (1) a growth/acute stage (between 7 and 21 days) and (2) a chronic stage (after 21 days) [33]. Longer AP induction periods (more than 21 days) could have allowed for an assessment of the chronic stage of AP [7]. A systemic condition counteracted by physical exercise was developed by performing AP induction on the left mandibular and maxillary first and second molars, considering that multiple periapical lesions in rats, that is, four teeth instead of two, can negatively impact systemic health [32].

Radiographic, micro‐CT and histopathological analyses were performed on distal root molars. It is important to highlight that, although the distal roots of a rat’s mandibular molars have a flattened root canal, thinner canal walls and a higher curvature compared to mesial roots [34], they can bring about a firmly established periapical lesion (AP) that can be measured, as shown in previous studies [30, 35].

In rodents, histopathological analysis is the gold standard to assess morphological changes in the bone and other tissues around the apex [31, 36]. However, this technique allows bone resorption analysis only on a single plane [36]. Micro‐CT analysis is a validated technique for three‐dimensional analysis of periapical bone resorption, since it provides multiplanar high‐resolution images [31]. Additionally, AP volume obtained by micro‐CT shows a high correlation with the AP area measured in histological sections [36].

The prescribed training protocol was for moderate‐intensity running (60% of the *T*_max_) on a treadmill [23, 28], which is an aerobic exercise (i.e., it depends on oxygen availability) that involves several large muscle groups. In rats, this type of moderate exercise, at 60% of the *T*_max_, promotes significant attenuation of IL‐6 and TNF‐α pro‐inflammatory markers [23]. One of the limitations of the present study is that it did not use different training intensities; however, we chose moderate‐intensity exercise because it has been shown to promote less oxidative stress in rats than low‐ and high‐intensity exercise [37]. In this sense, considering that AP is a consequence of an immuno‐inflammatory response that mediates the destruction of periapical tissues [2] and that physical activity promotes an anti‐inflammatory systemic effect [18, 19], we hypothesised that moderate physical exercise could impact AP development. This effect did not occur, since no differences were observed between the trained and sedentary groups with AP induction (S‐PA and Ex‐PA) in the histological, radiographical or micro‐CT analyses. These results differ from those of a recent study on rats, which revealed that the physical exercise group had lower AP volume than the sedentary group [23]. However, the authors applied a different AP induction time (28 days) and different duration of physical exercise (4 weeks), and the animals trained only after AP induction, not before, that is, they had no previous physical exercise training. Shorter durations, such as 7 days [38] or 14 days [39] can also be used, since it is not yet clear whether physical exercise is effective in the early or late stages of AP development. Additionally, different intensities and durations of physical exercise (i.e., a different training protocol) can also be considered. On the other hand, in the present study, although the moderate‐intensity aerobic training protocol was standardised for rats, caution is warranted when extrapolating these results to humans due to interspecies differences in metabolism, physiology and exercise scaling [40]. Our results also differ from those of a recent study that revealed smaller apical lesions and reduced inflammatory infiltrate in physically active rats compared to sedentary ones [26]. In that study, however, the animals underwent swimming training instead of treadmill running.

Previous studies in periodontics conducted on humans have suggested that physical exercise has positive effects on human periodontal health, such as a reduction in the prevalence of periodontal disease, and in pro‐inflammatory biomarkers [20, 21]. However, these studies are cross‐sectional, and cannot reveal causation or establish the direct effect of physical exercise on periodontal disease. In contrast, a recent study on humans revealed that physical exercise does not significantly affect the risk of developing periodontal disease [41].

From another perspective, we hypothesised that AP could impact physical exercise capacity negatively; however, this effect was not observed. It is difficult to draw a proper comparison, since this is the first study that has assessed this topic. Concerning humans, although it has been shown that oral inflammations, such as periodontal disease, impact the performance of athletes negatively, there are still no conclusive data regarding this effect, since there are no longitudinal studies [42]. For instance, a study showed that periodontal disease may be a risk factor for poor physical fitness in males [43]. However, the main limitation of the referenced cross‐sectional study was the same as that discussed above. In elderly people, oral problems (such as fewer remaining teeth, poor pocket depth, loss of attachment, etc.) are associated with lower physical activity performance. However, the evidence is still scarce, and more research is needed [44]. A study involving the ’oral inflammatory burden’, that is, a combination of periodontal and endodontic disease load, showed that the number of teeth with AP and/or root canal treatment in periodontal patients was associated independently with poor physical fitness in males [13]. However, the study did not include the ’no exercise’ group, and the imaging evaluation was two‐dimensional, that is, only radiographs were used. More research addressing parameters, such as different AP induction periods and different training protocols is needed to further the topic.

Animal models in endodontics are essential to ensure the validation, safety, biocompatibility and toxicology of new therapeutic alternatives in treating AP. However, specifically in the case of research in rodents, the main limitation in that such animals do not have a metabolism and pathophysiology similar to that of humans [40]. For this reason, animal models cannot correlate directly to human models or health‐related implications; therefore, the results must be interpreted with caution [45]. Lastly, the present study was performed using variables that may be delineated differently in future research, for example, AP induction time, duration and intensity of physical exercise prescription and response to root canal therapy in the model.

Overall, the results of the present study, within its limitations, show that there is no bidirectional relationship between physical exercise and AP; however, this effect may not be the same in humans, given their different biology, physiology and response to injuries. This requires further investigation using different models and study designs.

## 5. Conclusion

Physical exercise did not influence AP development, nor did AP affect physical exercise capacity in trained rats. However, these findings should be interpreted with caution, as this study represents an exploratory animal model. Further research is needed to validate these results in both animal and human studies, assessing different parameters, such as training type or varying periods of AP induction to better elucidate the relationship between physical exercise and AP.

## Ethics Statement

The experimental procedures of the present study were approved by the Animal Ethics Committee of the school of dentistry (Approval No. 20/2022).

## Conflicts of Interest

The authors declare no conflicts of interest.

## Author Contributions

Conceptualisation: Gisele Faria and Sandra Lia do Amaral. Data curation: Hernán Coaguila‐Llerena, Elda Olivia Nobre de Souza, Ana Beatriz Bardasi Solcia and Gilmara Gomes de Assis. Formal analysis: Hernán Coaguila‐Llerena, Elda Olivia Nobre de Souza, Ana Beatriz Bardasi Solcia, Gilmara Gomes de Assis, Sandra Lia do Amaral and Gisele Faria. Funding acquisition: Hernán Coaguila‐Llerena and Gisele Faria. Investigation: Hernán Coaguila‐Llerena, Gilmara Gomes de Assis, Sandra Lia do Amaral and Gisele Faria. Methodology: Hernán Coaguila‐Llerena, Elda Olivia Nobre de Souza, Ana Beatriz Bardasi Solcia, Gilmara Gomes de Assis, Paulo Sérgio Cerri and Gisele Faria. Project administration, supervision: Gisele Faria. Resources: Paulo Sérgio Cerri and Gisele Faria. Software: Hernán Coaguila‐Llerena, Paulo Sérgio Cerri and Gisele Faria. Validation: Hernán Coaguila‐Llerena, Gilmara Gomes de Assis, Sandra Lia do Amaral, Paulo Sérgio Cerri and Gisele Faria. Visualisation: Hernán Coaguila‐Llerena, Elda Olivia Nobre de Souza, Ana Beatriz Bardasi Solcia, Gilmara Gomes de Assis, Sandra Lia do Amaral, Paulo Sérgio Cerri and Gisele Faria. Writing – original draft, writing – review and editing: Hernán Coaguila‐Llerena, Ana Beatriz Bardasi Solcia, Elda Olivia Nobre de Souza, Sandra Lia do Amaral, Paulo Sérgio Cerri and Gisele Faria.

## Funding

This study was supported by the Deanship of Research (PROPe)/Deanship of Postgraduate Studies (PROPG) of Sao Paulo State University (UNESP) (Grant 04/2022) and the Fundação de Amparo à Pesquisa do Estado de São Paulo (FAPESP) (Grants 23/12023‐7 and 23/11282‐9).

## Data Availability

The data used to support the findings of this study are available from the corresponding author upon request.
